# lncRNA SNHG9 Promotes Cell Proliferation, Migration, and Invasion in Human Hepatocellular Carcinoma Cells by Increasing GSTP1 Methylation, as Revealed by CRISPR-dCas9

**DOI:** 10.3389/fmolb.2021.649976

**Published:** 2021-04-09

**Authors:** Shanting Ye, Yong Ni

**Affiliations:** ^1^Graduate School of Guangzhou Medical University, Guangzhou, China; ^2^Department of Hepatobiliary Surgery, Shenzhen Second People’s Hospital, Shenzhen, China

**Keywords:** SNHG9, hepatocellular carcinoma, CRISPR-dCas9, GSTP1, promoter methylation

## Abstract

Hepatocellular carcinoma (HCC) is among the major causes of cancer-related mortalities globally. Long non-coding RNAs (LncRNAs), as epigenetic molecules, contribute to malignant tumor incidences and development, including HCC. Although LncRNA SNHG9 is considered an oncogene in many cancers, the biological function and molecular mechanism of SNHG9 in HCC are still unclear. We investigated the effects of lncRNA SNHG9 on the methylation of glutathione S-transferase P1 (GSTP1) and the progression of HCC. Histological data analysis, CRISPR-dCas9, and cytological function experiment were used to study the expression level and biological function of SNHG9 in HCC. There was an upregulated expression of SNHG9 in HCC, which was associated with shorter disease-free survival. Knockdown of SNHG9 can inhibit cell proliferation, block cell cycle progression, and inhibit cell migration and invasion by upregulating GSTP1. LncRNA SNHG9 recruits methylated enzymes (DNMT1, DNMT3A, and DNMT3B) to increase GSTP1 promoter methylation, a common event in the development of HCC. Inhibition of lncRNA SNHG9 demethylates GSTP1, which prevents HCC progression, presents a promising therapeutic approach for HCC patients.

## Introduction

Hepatocellular carcinoma (HCC) is a malignant tumor originating from hepatocytes and is the most common primary hepatic malignant tumor. HCC is the sixth most common cancer and the second-leading cause of tumor-related mortalities globally (Zhu et al., 2016; Kim et al., 2017; Zhu and Rhim, 2019). China accounts for approximately 55% of the newly diagnosed HCC cases, attributable to high incidence rates of chronic hepatitis B virus (HBV) infections (Wang et al., 2017). Despite the improvements in clinical therapeutic efficacy, the recovery and long-term survival rate of HCC patients remain low (Shen et al., 2017; Umeda et al., 2019). Recently, the discovery of targeted therapies has presented novel opportunities for treating liver cancer (Chen et al., 2019; Fu and Wang, 2019); however, the few HCC targeted drugs available are often associated with resistance. Therefore, understanding the pathogenesis of HCC can reveal potential therapeutic targets, which may facilitate the development of novel molecular targeted therapies.

Long non-coding RNAs (lncRNAs) are newly discovered non-protein-coding RNA molecules composed of over 200 nucleotides (Chen et al., 2016; Mathy and Chen, 2017). They are found in the nucleus or cytoplasm and have complex structures that transmit a variety of cellular functions and play an important role in a variety of diseases (Beermann et al., 2016; Chen et al., 2017). There is increasing evidence that lncrNA expression disorders affect gene regulation and promote cancer development (Fang and Fullwood, 2016; Tang et al., 2017; Arun et al., 2018). Small nucleolar RNA host gene 9 (SNHG9) is a membrane-lipid-related LncRNA that has been shown to regulate the proliferation of pancreatic cancer cells (Zhang et al., 2018) and glioblastoma cells (Zhang et al., 2019). However, the role of SNHG9 in HCC remains unclear.

Glutathione s-transferase P1 (GSTP1) catalyzes the Q coupling of electrophilic molecules to glutathione, is involved in detoxification (Crawford and Weerapana, 2016; Gurioli et al., 2018). Research shows that GSTP1 gene polymorphism is associated with HCC among other cancers (Wang et al., 2016; Chatterjee and Gupta, 2018; Ding et al., 2019). Besides, the methylation of the GSTP1 promoter is correlated with gene expression and function (Sophonnithiprasert et al., 2020).

We sought to establish the role of SNHG9 and GSTP1 in HCC pathology. The findings indicated the upregulated expression of SNHG9 in HCC is associated with shorter disease-free survival. Upregulating GSTP-1 expression led to cell proliferation inhibition, cell cycle progression blockage, cell migration, and invasion inhibition. LncRNA SNHG9 recruits methylated enzymes (DNMT1, DNMT3A, and DNMT3B) to promote the GSTP1 promoter’s methylation, which is a common event in HCC development. Therefore, we underscore the molecular mechanisms of HCC pathogenesis and potential targets for treatment.

## Materials and Methods

### Clinical Specimens

In this study, HCC specimens and adjacent tissues were obtained from patients from the First Affiliated Hospital of Shenzhen University from 2015 to 2018. All patients received written informed consent. The research was approved by the Ethics and Human Sciences Committee of Shenzhen University and conducted as per the Helsinki Declaration.

### Public Data Set Download

Liver cancer genome atlas (TCGA) level 3 RNA sequencing—seq (RNA) data and their medical data were derived from the genomic data sharing (GDC) data portal website^1^. Also, we obtained three datasets (GSE19915 GSE13507 GSE3167) from Gene Expression Omnibus (GEO^2^) based on the microarray data sets (Zhang et al., 2018, 2019).

### Real-Time Quantitative Polymerase Chain Reaction

Total RNA was extracted using Trizol reagent (Invitrogen) as per the manufacture’s instructions. cDNA was reverse-transcribed to 2–6 g total RNA using M-MLV reverse transcriptase (Promega, Madison, WI, United States). Real-Time Quantitative Polymerase Chain Reaction (Rt-qPCR) was performed by the StepOnePlus real-time PCR system (ABI, United States) using a 20 L reaction mixture consisting of 0.1 M primers, 10 L 2 × FastStart Universal SYBR Green Master (Rox, Switzerland), and 20–100 ng cDNA samples. All the experiments were repeated at least three times. Relative mRNA levels were standardized to -actin mRNA levels, using 2^–ΔΔ*CT*^ Methods.

### CCK-8 and Clone Formation Tests

About 1 × 10^4^ cells were inoculated into 96-well plates overnight. CCK-8 reagent (Japanese Dojindo) was mixed with 10%FBS + RMI-1640 medium at a ratio of 1:9 and cultured in the dark for 3 h. Absorbance was measured after 0, 24, 48, and 72 h at 450 nm using a multi-tablet reader (Bio-RAD, United States). The ratio of absorbance after 24, 48, and 72 h to absorbance at 0 h was used to investigate the cells’ proliferation ability in five independent experiments.

In the clone formation experiment, 200, 400, and 800 cells were cultured in a 6-well plate at 37°C until the clone was visible to the naked eye. The clones were stained in 0.1% crystal violet and 20% methanol, after which they were imaged and counted.

### Cell Migration Test

Each group’s logarithmically growing cells were seeded into a 6-well plate (1 × 10^6^ cells/well). A line was evenly drawn on the back of the 6-well plate using a marker pen as the subsequent photo position and recorded. When the cells were attached to the plate surface, the old medium was replaced with a fresh culture medium containing 1% fetal bovine serum (FBS), after which the cells were then starved for 12 h. The point of a 200 L spear was scraped out in a straight line using a ruler on the board. Then the cells were washed three times with 2 ml PBS and photographed at 0 h and 24 h. The experiment was replicated thrice. In each image, 15 lines were uniformly distributed, and the distances across the lines were measured and averaged.

### Transwell Assay

Matrigel (BD Biosciences, San Jose, CA, United States) was added to a cell medium without serum (1:1 v/v). Matrigel was then polymerized in a Transwell chamber (Corning, NY, United States) at 50 L/well and placed in a 37°C incubator. The cells in the logarithmic growth phase were starved for 24 h in a cell culture medium containing 1% fetal bovine serum. After separation, the cells were resuspended in a 1 × 10^6^ serum-free medium. 50 L FBS was mixed with a 50 L medium containing 2% FBS and added to the apical chamber of the Transwell. A 600 L cell culture medium containing 10% fetal bovine serum was added to the basal lateral compartment and cultured in an incubator at 37°C and 5% CO_2,_ saturated humidity and sufficient oxygen for 24 h. The Transwell chamber was then removed, fixed with 4% paraformaldehyde, and stained with crystal violet. Finally, the invasion cells were counted in 5 randomly selected fields under an inverted microscope. Three independent experiments were conducted, and the average value was taken.

### Cell Cycle Analysis (Image-Flow Cytometry)

We harvested the transfected cells, washed them twice with cold PBS, and then fixed them with 70% cold ethanol. A cell cycle analysis kit (MultiSciences, China) was used to determine cell cycle distribution. ModFit 5.2 software was used to sequence and calculate cells at different stages of the cell cycle.

### Methylation-Specific Polymerase Chain Reaction

The DNA of cells was extracted using a standard procedure. The cells were treated with protease K, after which chloroform was added. The concentration of the extracted DNA was determined using a spectrophotometer. Sodium bisulfite was mixed with the DNA extract to produce vulcanized DNA. The PCR products were then electrophoresed by agarose gel, and the target bands were observed by gel imager.

### Double Luciferase Reporter Gene Detection

The GSTP1 promoter and lncRNA SNHG9 full-length sequences were derived from the NCBI database^3^. Four plasmid expression vectors (HA-PCMV5-GSTP1, PCMV5-SNHG9, GSTP1 promoter—LUC, and pCMV5 (control) were constructed from Addgene (Cambridge, MA, United States), including. Then the GSTP1 promoter—LUc and pCMV5 vectors, HA-PCMV5-GSTP1, GSTP1 promoter—Luc, and PCMV5-SNHG9 vectors, were transfected into the cells, respectively. Luciferase activity was then determined using the luciferase reporter assay kit (Promega, Madison, WI, United States). Briefly, 20 L of cell lysate was added to a 1.5 mL centrifuge tube followed by 100 L of LARII solution. After mixing, the absorbance was measured at 460 nm with a luminescence detector (Promega, Madison, WI, United States).

### Fluorescence *in situ* Hybridisation

The Fluorescence *in situ* Hybridisation (FISH) technique was performed using the RiboTM lncRNA FISH Probe Mix (Red) to detect the localization of SNHG9 in HCC cells using the procedure described by the manufacturer (Guangzhou RiboBio Co., Ltd., Guangzhou, Guangdong China). Briefly, the cells were inoculated in a six-well culture plate and covered with slides. The cells were 80% confluent after 24 h of culture. The glass was washed with PBS, fixed with 1 mL 4% paraformaldehyde at room temperature, and treated with protease K (2 g/mL), glycine, and acetylation reagent. Subsequently, the additive plate was used with a 250 L prehybridization solution and incubated at 42°C for 1 h. After removing the prehybridization solution, the 250 L hybridization solution consists of an overnight incubation of a detector (300 ng/mL) at 42°C, followed by three and phosphate buffer brine washing Tween-20 (PBST). Then, the nuclei were diluted with PBST for 4′ and stained with 6-diamino-2-pheninindole (DAPI) (1:800). 24-well plates were added for 5 min and washed with PBST for 3 min. Finally, the cells were observed under a fluorescence microscope (Olympus Optical Co., Ltd., Tokyo, Japan).

### RNA Binding Protein Immunoprecipitation

The binding analysis of lncRNA SNHG9 with DNMT1, DNMT3A, and DNMT3B was carried out step by step according to the instructions of RNA Binding Protein Immunoprecipitation (RIP) Kit (Millipore, Billerica, MA, United States). Briefly, cells were washed using pre-cooled phosphate-buffered saline (PBS) for 5 min. with 25 eases to L Tris–Hcl (pH 7.5),150 eases to L potassium chloride, and 2 eases to L ediamine tetraacetic acid (EDTA),0.5% NP40 L was more easily associated with sodium fluoride,1 was more easily associated with Deloitte,100 U/ml RNasin ribozyme inhibitor and EDTA-free protease inhibitor, and centrifuged at 14,000 rpm for 10 min at 4°C. A cell extract portion was then taken as an input, and the other part was incubated with an antibody for co-precipitation. To sum up, 50 L magnetic beads were extracted from each co-precipitation reaction system. After washing, they were resuspended in 100 L RIP washing solution, and then 5 g antibody was added in groups for incubation and combination. Subsequently, the magnetic bead—antibody complex was washed and again suspended in 900 L RIP wash solution and incubated at 4°C overnight with 100 L cell extract. The samples were then placed on a magnetic base to collect globin complexes. Samples and inputs were separated by protease K, RNA was extracted, and quantitative polymerase chain reaction (RT-QPCR) was performed. In this study, rabbit anti-human antibodies DNMT1 (1:100, AB13537), DNMT3A (1:100, AB2850), and DNMT3B (1:100, AB2851) were uniformly mixed for 30 min at room temperature. Rabbit anti-human immunoglobulin G (IgG) (1:100, AB109489) was used as the negative control (NC). The antibodies were sourced from Abcam (Cambridge, United Kingdom).

### Chromatin Immunoprecipitation

After attaining a 70–80% confluence, cells in each group were harvested and fixed in 1% formaldehyde for 10 min at room temperature to cross-link DNA and proteins. The DNA and proteins were then randomly separated by ultrasound. The cells were centrifuged at 4°C, 13,000 × *g*. The supernatant was mixed with rabbit anti-IgG (AB109489 1:100, Abcam Inc., Cambridge, United Kingdom) CNC antibody and the target protein-specific antibodies (DNMT1 (AB13537 1:100, Abcam Inc., Cambridge, United Kingdom), DNMT3A (AB2850 1:100, Abcam Inc., Cambridge, United Kingdom), and DNMT3B (Abcam AB2851, 1:100, Cambridge, United Kingdom), followed by overnight incubation at 4°C. The endogenous DNA-protein complex was then precipitated with protein agarose/agarose. After centrifugation for a while, the supernatant was removed, and the non-specific complex was rinsed. Then, the cross-linking was completed overnight at 65°C, and the DNA fragments were extracted and purified with phenol/chloroform solution. Enrichment of GSTP-1 promoter fragment bound to DNMT1, DNMT3A, and DNMT3B was detected by gSTP-1 promoter fragment specific primer.

### Statistical Analysis

Data analysis was performed using SPSS 20.0 software. The quantitative variables were summarized as mean ± standard deviation (SD). The students’ *T*-test was used to analyze the differences between the two independent groups. The two-tailed test was considered significant when *P* < 0.05 was calculated.

## Results

### SNHG9 Expression Was Upregulated in HCC Tissue Samples

**FIGURE 1:**
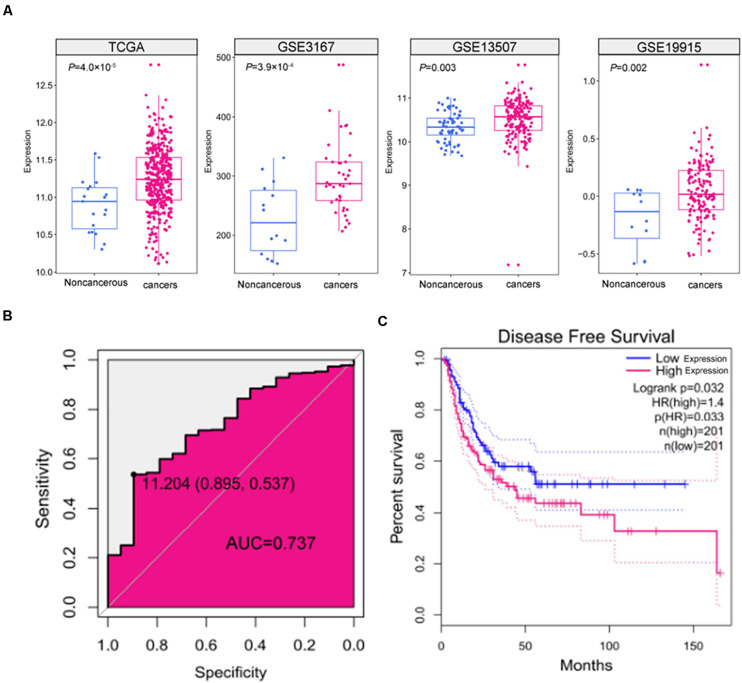
The expression and clinical significance of SNHG9 in HCC. **(A)** SNHG9 expression in non-cancerous liver tissues and HCC samples, based on data from the public dataset. **(B)** ROC curve and AUC of SNHG9. **(C)** Kaplan–Meier survival curves of the two groups of patients based on TCGA data. Red: Patients with high SNHG9 expression. Blue: Patients with low expression of SNHG9.

To assess the expression of SNHG9 in HCC samples, we downloaded 408 HCC sample and 19 adjacent normal tissue TCGA data. TCGA data showed that SNHG9 was significantly upregulated in HCC (*P* = 4.0 × 10^–5^). GEO datasets showed significant increases in SNHG9 in HCC samples (*P* = 3.92 × 10^–4^ for GSE3167, *P* = 0.003 for GSE13507, and *P* = 0.002 for GSE19915) (Figure 1A). The Receiver Operating Characteristic (ROC) curve was used to analyze the sensitivity and specificity based on TCGA data to evaluate the diagnostic value of SNHG9 for HCC. The area under the SNHG9 curve (AUC) was 0.737. At the critical value of 11.20, the sensitivity and specificity were 0.54 and 0.90, respectively (Figure 1B). Survival analysis showed that high SNHG9 expression was associated with shorter disease-free survival (*P* = 0.032) (Figure 1C).

### Downregulation of the SNHG9 Gene Inhibited the Proliferation of Hepatocellular Carcinoma Cells and Blocked the Progression of the Cell Cycle

**FIGURE 2:**
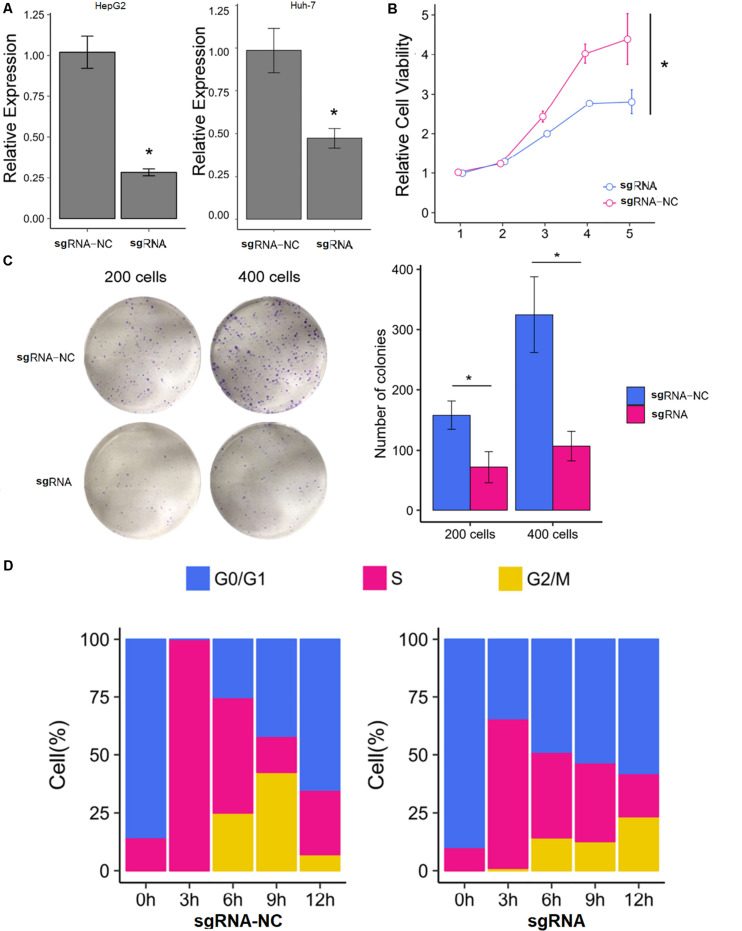
Knockdown of SNHG9 can inhibit the proliferation and cell cycle progression of HCC cells. **(A)** The expression of SNHG9 in sgRNA-NC and sgRNA-SNHG9 transfected cells as detected by RT-qPCR. (*n* = 3 independent experiments). **(B)** The CCK-8 method was used to evaluate the effect of silencing SNHG9 on cell proliferation. (*n* = 3 independent preparations) *P < 0.05. **(C)** Clones are stained with Giemsa and photographed with a digital camera. The clone number was calculated and statistically analyzed. **(D)** The cell cycle was synchronized with the use of a double thymine block. The stack diagram shows the percentage of cells in each cycle at each time point. Left: Cells transfected with sgRNA-NC. Right: Cells transfected with sgRNA-SNHG9. Blue: Phase G0. Red:S phase. Gold:G2 and M phase.

The HepG2 and Huh-7 HCC cell lines were established to effectively express SNHG9 targeting sgRNA (sgRNA-SNHG9) and negative control sgRNA (sgRNA-NC), respectively. Rt-qPCR and Western blot were used to verify the effect of knockout. After transfection of sgRNA-SNHG9, the expression of SNHG9 was significantly decreased (HEPG2: *P* = 0.009; Huh-7: *P* = 0.003; Figure 2A). Compared with the control group, the relative expression of lncRNA in HepG2 and Huh-7 cells was reduced by 72.2 and 50.3%, respectively. These results indicated that SNHG9 was effectively knocked out. We compared the proliferation of sgRNA-SNHG9 and sgRNA-NC groups in HEPG2 cells using the CCK-8 method to determine the role of SNHG9 in HCC cells. SNHG9 knockdown significantly reduced cell proliferation (*P* = 0.018) (Figure 2B). On day 5, the proliferation of the sgRNA-SNHG9 group decreased by 1.57 ± 0.95. In the clone formation experiment, we found that the clonal formation capacity of Huh-7 cells was significantly inhibited than the negative control cells (Figure 2C).

We dynamically compared the cell cycle progression of the sgRNA-SNHG9 group and sgRNA-NC group in HEPG2 cells to establish the function of SNHG9 in the cell cycle. Cell cycle synchronization was first blocked by double thymine at the G1/S boundary and then released simultaneously. We found that most cells in the sgRNA-NC group were in the G0/G1 phase and entered the G2/M phase through the S phase within 6–12 h. In the sgRNA-SNHG9 group, the G0/G1 phase cells slowly entered the S phase from 0 to 12 h, suggesting that SNHG9 knockdown led to cell cycle arrest at the G0/S phase (Figure 2D). These results indicated that silencing lncRNA SNHG9 could impede HCC cell growth.

### Downregulation of SNHG9 Inhibited the Migration and Invasion of Hepatocellular Carcinoma Cells

**FIGURE 3:**
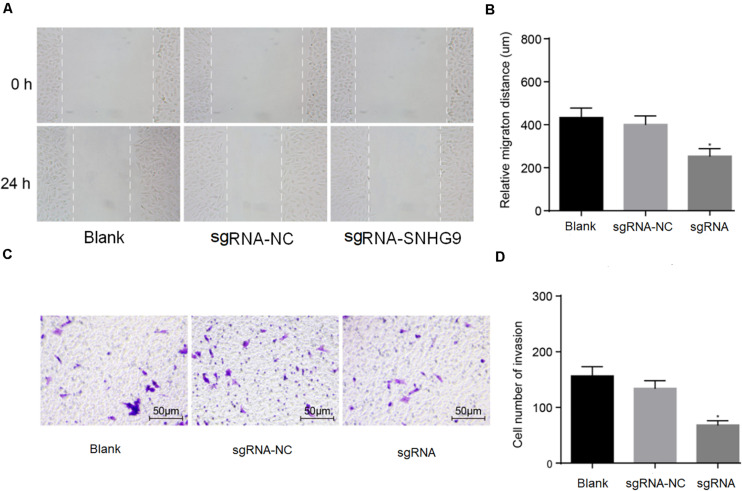
The downregulation of SNHG9 inhibited the migration and invasion of HCC cells. Panles **(A,B)** were used to detect the cell migration after SNHG9 knockdown by scratch test. **(C,D)** Cell invasion after SNHG9 gene knockout was detected by Transwell method. Statistical data were presented as mean ± standard deviation, and One-Way ANOVA was used. The experiment was repeated three times; *P < 0.05.

The scratch test showed that cell mobility was significantly reduced after sgRNA-SNHG9 treatment (*P* < 0.05; Figures 3A,B). The results suggest that silencing lncRNA SNHG9 can inhibit cell migration. Subsequently, Transwell’s results showed that sgRNA-SNHG9 significantly reduced cell invasion (*P* < 0.05; Figures 3C,D), indicating that the cell invasion rate could be inhibited after lncRNA SNHG9 was inhibited.

### LncRNA SNHG9 Promotes DNA Methylation of the GSTP1

**FIGURE 4:**
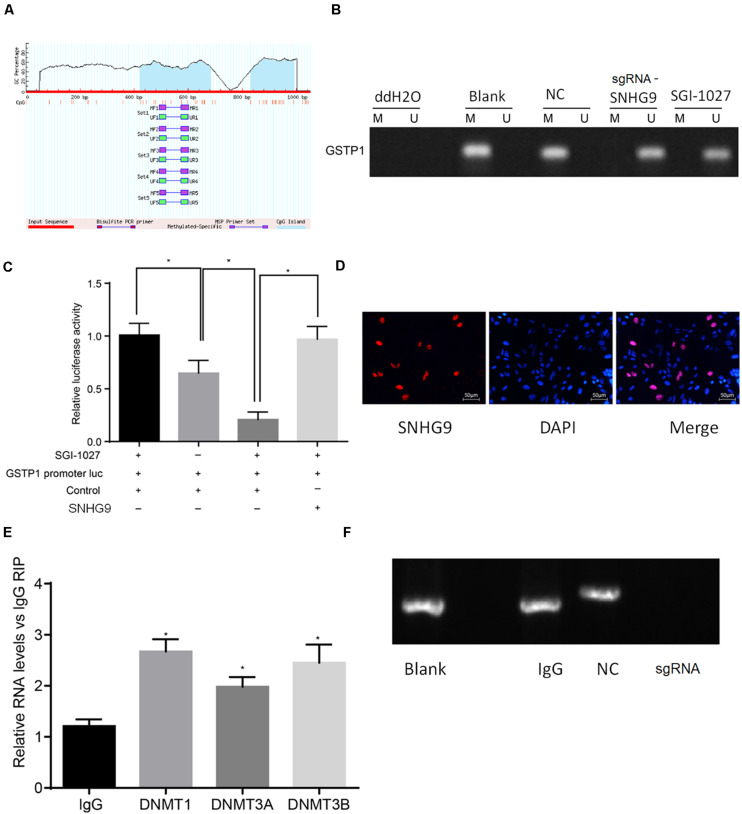
SNHG9 promotes GSTP1 promoter methylation by recruiting methylated proteins. **(A)** localization of GSTP1 methylated SNHG9 and CpG Island predicted by bioinformatics; **(B)** Ms-PCR detected the methylation level of GSTP1 promoter after sgRNA-SNHG9 and SGI-1027 treatment. **(C)** Double luciferase reporter gene assay confirmed the binding of SNHG9 to GSTP1 promoter. **(D)** FISH analysis of lncRNA SNHG9; The RIP analysis of panel **(E)**, SNHG9 combined with DNMT1, DNMT3A, and DNMT3B; CHIP detection of GSTP1 promoter in panel **(F)**, IgG, and sgRNA-SNHG9.

Bioinformatics analysis showed that lncRNA SNHG9 was located in the nucleus, and online CpG prediction software was used to predict the presence of methylated CpG islands in the GSTP1 promoter (Figure 4A). The Methylation-Specific Polymerase Chain Reaction (mS-PCR) results showed that after treatment with sgRNA-SNHG9 and SGI-1027, methylation inhibitors of GSTP1, the GSTP1 promoter’s methylation degree was significantly reduced (Figure 4B). The results of double luciferase reporter gene detection (Figure 4C) showed that luciferase activity of GSTP1 promoter co-transfected with GSTP1 gene was significantly lower than that of GSTP1 promoter transfected with GSTP1 gene only (*P* < 0.05). The luciferase activities of the GSTP1 promoter, GSTP1 gene, and lncRNA SNHG9 were significantly lower than that of GSTP1 promoter after co-transfection with GSTP1 gene (*P* < 0.05). Subsequently, FISH results showed that lncRNA SNHG9 was mainly located in the nucleus (Figure 4D). Besides, RIP analysis showed that lncRNA SNHG9 targeted and bound methylation-related proteins (DNMT1, DNMT3A, and DNMT3B) compared with the IgG group (*P* < 0.05) (Figure 4E). CHIP analysis results revealed significantly higher enrichment of the blank group’s GSTP1 promoter region than the sgRNA-SNHG9’s group (Figure 4F). These findings provide evidence that lncRNA SNHG9 inhibits the expression of GSTPI by promoting the methylation of the GSTP1 promoter.

### Downregulation of SNHG9 Inhibits Proliferation, Migration, and Invasion of HCC Cells by Upregulating GSTP1

**FIGURE 5:**
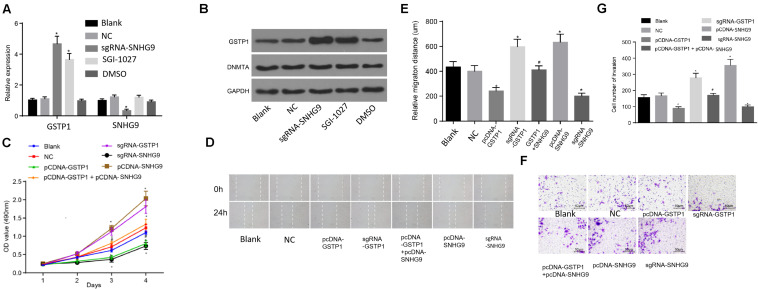
Knockdown of SNHG9 can inhibit the proliferation, migration, and invasion of HCC cells by upregulating GSTP1. **(A)** The expression of SNHG9 and GSTP1 after sgRNA-SNHG9 and SGI-1027 treatment and detected by RT-qPCR; **(B)** Western blot analysis of GSTP1 and DNMT after treatment with sg-SNHG9 and SGI-1027; **(C)** The proliferation of hepatocellular carcinoma cells treated with sgRNA-SNHG9, sgRNA-GSTP1, PCDNA-GSTP1, PCDNA-SNHG9, and PCDNA-GSTP1 + SNHG9 as detected by the CCK-8 method. Panels **(D,E)** were used to detect the migration of hepatocellular carcinoma cells treated with sgRNA-SNHG9, sgRNA-GSTP1, PCDNA-GSTP1, PCDNA-SNHG9, and PCDNA-GSTP1 + SNHG9 by the scratch method. **(G)** Transwell assay was used to detect the invasive ability of sgRNA-SNHG9, sgRNA-GSTP1, PCDNA-GSTP1, PCDNA-SNHG9, and PCDNA-GSTP1 + SNHG9 to treat liver cancer cells.

The RT-qPCR was used to detect the expression of GSTP1 mRNA after treatment with sgRNA-SNHG9, sgRNA-GSTP1, PCDNA-GSTP1, PCDNA-SNHG9, and PCDNA-GSTP1 + SNHG9. The results are presented in Figure 5A. The GSTP1 mRNA’s expression after treatment with PCDNA-GSTP1 and sgRNA-SNHG9 was significantly higher (*P* < 0.05). The expression of GSTP1 mRNA after PCDNA-GG9 +, sgRNA-GSTP1, and PCDNA-SNHG9 treatment was significantly decreased (*P* < 0.05) compared with that of the PCDNA-GSTP1 treatment. The expression of lncRNA SNHG9 after PCDNA-SNHG9 treatment was significantly increased (*P* < 0.05), while the expression of lncRNA SNHG9 after sgRNA-SNHG9 treatment was significantly low (*P* < 0.05). After treatment with sgRNA-GSTP1 and PCDNA-GSTP1, the expression of lncRNA SNHG9 showed no significant change (*P* > 0.05). Western blot analysis showed significantly higher GSTP1 protein level after PCDNA-GSTP1 and sgRNA-SNHG9 treatment (*P* < 0.05), while the GSTP1 protein level decreased significantly after treatment with sgRNA-GSTP1 and PCDNA-SNHG9 (*P* < 0.05). The GSTP1 protein level was significantly lower after PCDNA-GSTP1 + SNHG9 treatment than in PCDNA-GSTP1-treated group (*P* < 0.05; Figure 5B). CCK8 experimental results showed that the proliferation of the cells on the third and fourth day after the treatment of PCDNA-GSTP1 and sgRNA-SNHG9 was significantly low (*P* < 0.05), while the proliferation of the cells on the third and fourth day after the treatment of sgRNA-GSTP1 and PCDNA-SNHG9 was significantly high (*P* < 0.05). The cell proliferation after PCDNA-GSTP1 + SNHG9 treatment was significantly higher than that after PCDNA-GSTP1 treatment (*P* < 0.05) (Figure 5C). Besides, the scratch test revealed significantly reduced cell migration after PCDNA-GSTP1 and sgRNA-SNHG9 treatment (*P* < 0.05), while the cell migration was significantly enhanced after treatment with sgRNA-GSTP1 and PCDNA-SNHG9 (*P* < 0.05). PCDNA-GSTP1 + SNHG9 indicated significantly higher cell migration than PCDNA-GSTP1 (*P* < 0.05) (Figures 5D,E). Subsequently, Transwell assay results showed that pcDNA-GSTP1 and sgRNA-SNHG9 significantly reduced cell invasion (*P* < 0.05), while sgRNA-GSTP1 and PCDNA-SNHG9 significantly increased cell invasion (*P* < 0.05). The PCDNA-GSTP1 + SNHG9-triggered cells were significantly more aggressive than the PCDNA-GSTP1-triggered ones (*P* < 0.05; Figures 5F,G). In summary, lncRNA with low expression of SNHG9 and high expression of GSTP1 deters metastasis.

## Discussion

Elucidating the molecular mechanisms of hepatocellular carcinoma remains an urgent clinical challenge. Currently, the therapeutic efficacy of available drugs for advanced liver cancer is limited (Faivre et al., 2020; Pfister et al., 2020). In recent years, lncRNA has aroused extensive research interest in various countries in the world. There is increasing evidence that some lncRNAs are associated with the incidence and development of cancer. Multiple lncRNAs have been identified as playing a key role in HCC (Huang et al., 2020; Wei et al., 2020; Yang et al., 2020). LncRNAs may play an important role in predicting prognosis, treatment response, and cancer recurrence based on the patients’ genetic profiles and can be used in a personalized treatment design. In addition to editing and modifying DNA sequences, the CRISPR system has also been proven to regulate gene transcription, which brings unprecedented convenience to the study of lncRNA functions. The dCas9 protein could inhibit lncRNA expression by blocking the binding of RNA polymerase and promoters through a roadblock effect (Zhou et al., 2018; Huynh et al., 2020).

SNHG9 has been identified as an oncogene in pancreatic cancer and glioblastoma (Zhang et al., 2018, 2019), but its function is unclear. This study attempted to explore the biological function of SNHG9 in HCC. We investigated the expression level of SNHG9 in HCC clinical specimens. Clinical data revealed overexpression of SNHG9 in HCC tissues than in normal counterparts, and high SNHG9 expression may indicate poor prognosis. Then, we investigated the biological function of SNHG9 using CRISPR-dCas9. Unlike other classical gene silencing methods, such as RNAi, which inhibit gene expression by degrading mRNA in the cytoplasm, the CRISPR-dcas9 system works as a transcriptional suppressor at the DNA level. The binding of dCas9 protein to the target can interfere with RNA polymerase binding in space and play a role in inhibiting the transcription of targeted genes, a process known as CRISPRi (or CRISPR interference). We found that reducing the expression of SNHG9 could inhibit HCC metastasis. Moreover, we investigated the molecular mechanism of SNHG9 in HCC. The binding of SNHG9 to DNA methyltransferase was observed, and the knockout of SNHG9 may cause GSTP1 promoter demethylation, depicting its role in gene expression and tumorigenesis.

In summary, these findings present new insights on the importance of SNHG9 in HCC progression and offer a theoretical basis for developing lncRNA-based targeted therapies for HCC.

## Data Availability Statement

The original contributions presented in the study are included in the article/supplementary material, further inquiries can be directed to the corresponding author/s.

## Ethics Statement

The research was approved by the Ethics and Human Sciences Committee of Shenzhen University and conducted in accordance with the Helsinki Declaration. All patients received written informed consent.

## Author Contributions

SY: interpretation or analysis of the data, preparation of the manuscript, and revision for important intelectual content. YN: conception and supervision. Both authors contributed to the article and approved the submitted version.

## Conflict of Interest

The authors declare that the research was conducted in the absence of any commercial or financial relationships that could be construed as a potential conflict of interest.

## Footnotes

1 https://portal.gdc.cancer.gov/

2 http://www.ncbi.nlm.nih.gov/geo/

3 https://www.ncbi.nlm.nih.gov/gene
